# Pelvic flexion/extension and its impact on functional acetabular alignment and stability following total hip replacement

**DOI:** 10.1007/s00264-023-06060-w

**Published:** 2023-12-19

**Authors:** Mahmoud A Hafez, Branislav Jaramaz, Anthony M DiGioia

**Affiliations:** 1 The Orthopaedic Department, October 6 University, Giza, Egypt; 2 Smith and Nephew Robotics, Pittsburgh, PA USA; 3 The Bone and Joint Center, Magee Women’s Hospital of the University of Pittsburgh Medical Center, Pittsburgh, PA USA

**Keywords:** Pelvic flexion/extension, Total hip replacement, Impingement, Classification system, Spinopelvic alignment, Hip dislocation, Instability

## Abstract

**Purpose:**

Dislocation following total hip arthroplasty (THA) remains a significant clinical problem and can occur even with optimal implant alignment. We hypothesized that different patterns of pelvic flexion/extension (PFE) during daily activities may influence acetabular alignment and contribute to impingement and instability following THA. Recently, there has been an increased interest in spinopelvic alignment and its impact on THA. Therefore, this study aimed to identify different patterns of PFE that could be predictive of instability following THA.

**Methods:**

A range of motion (ROM) simulator was used to demonstrate the effects of different patterns of PFE on ROM and impingement. The findings were applied to PFE measurements obtained from 84 patients in standing and sitting positions.

**Results:**

Three different categories of PFE were identified: normal, hypermobile, and stiff. ROM simulator revealed that changes in PFE had affected ROM and impingement significantly. Patients in the stiff pelvis category, even with “optimal” implant alignment, were more susceptible to implant impingement.

**Conclusions:**

The different patterns of PFE during daily activities could affect acetabular alignment and stability following THA. We propose a classification system that can identify different types of PFE and predict their effects on the stability of prostheses following THA. Hence, we believe that patients with unfavorable PFE may require modified cup alignment.

## Introduction

Dislocation is one of the most devastating complications that can occur after total hip arthroplasty (THA), and it is the second most common complication after aseptic loosening [1]. The rate of dislocation among those with primary THA is 0.2% to 10% and could reach as high as 28% after revision surgery [2]. In the USA, dislocation (17.3%) was the main indication for revision surgeries, followed by loosening (16.8%) [3]. According to one report, the rate of revision due to instability has increased from 6.2 to 10.7% between 1990 and 2000 [4]. Although the causes of dislocation are multifactorial, femoral neck fracture, osteonecrosis of the femoral head, previous surgery, prosthetic and/or bony impingement, and choice of the surgical approach are the major contributory factors [5–7].

It is believed that the malposition of the acetabular cup is a common surgical factor for dislocation; consequently, acetabular orientation is of crucial importance for THA stability [8–10]. Excessive anteversion or retroversion of the femoral component can lead to instability, where anteversion can lead to dislocation in the extension and external rotation position while internal rotation leads to dislocation in the case of femoral retroversion [11]. However, dislocation can still happen even with apparent optimal implant alignment [12]. During surgery, the acetabular cup is aligned while the patient is in the supine or lateral position, but dislocation usually occurs when the patient moves while standing or sitting [13].

Few studies identified the variability in pelvic flexion/extension (PFE) during different body positions [14–16], and some authors questioned whether this variability could affect acetabular alignment [11]. Several authors examined the variability of PFE during activities of daily life (ADL) [14, 15]. Siebenrock et al. reported the effects of pelvic tilting on acetabular retroversion and its contribution to the measurement errors during the assessment of acetabular alignment and its importance in preoperative planning of reorientation osteotomy of the acetabulum [14]. Hyodo et al. found that some of the ADL such as crouching or putting on pants could have a greater angle of flexion than other ADL as walking or climbing stairs [15]. Eddine et al. reported that up to 20° errors might occur while measuring cup anteversion in the supine position if no account was made for the pelvic tilting [16]. Few cases of unstable THA have been reported in the literature to be due to abnormal flexion/extension of the pelvis [17, 18]. And more discussions about the subject were published in the past couple of years [19–29].

Computerized tomography (CT) scan is commonly used to measure PFE [30]. The modified Thomas test (MTT) was also suggested to be used as a measure for hip extension, yet controlling pelvic tilt is a must for the test to be a reliable tool [31]. Eckman et al. validated the use of lateral pelvic radiographs in measuring PFE [32]. In a previous study, we measured PFE in standing and sitting positions in 84 patients using lateral pelvic radiographs [33]. The anterior pelvic plane (APP) defined by the anterior superior iliac spines (ASIS) and pubic tubercles was used as a reference to measure PFE in the sagittal plane. The results showed substantial variations of PFE during standing and sitting for a patient and between patients. However, the clinical significance of this individual variation and its relation to the stability in THA has not been confirmed.

This study aimed to identify different patterns of PFE and to determine their effects on impingement and stability following THA.

## Methods

**Fig. 1:**
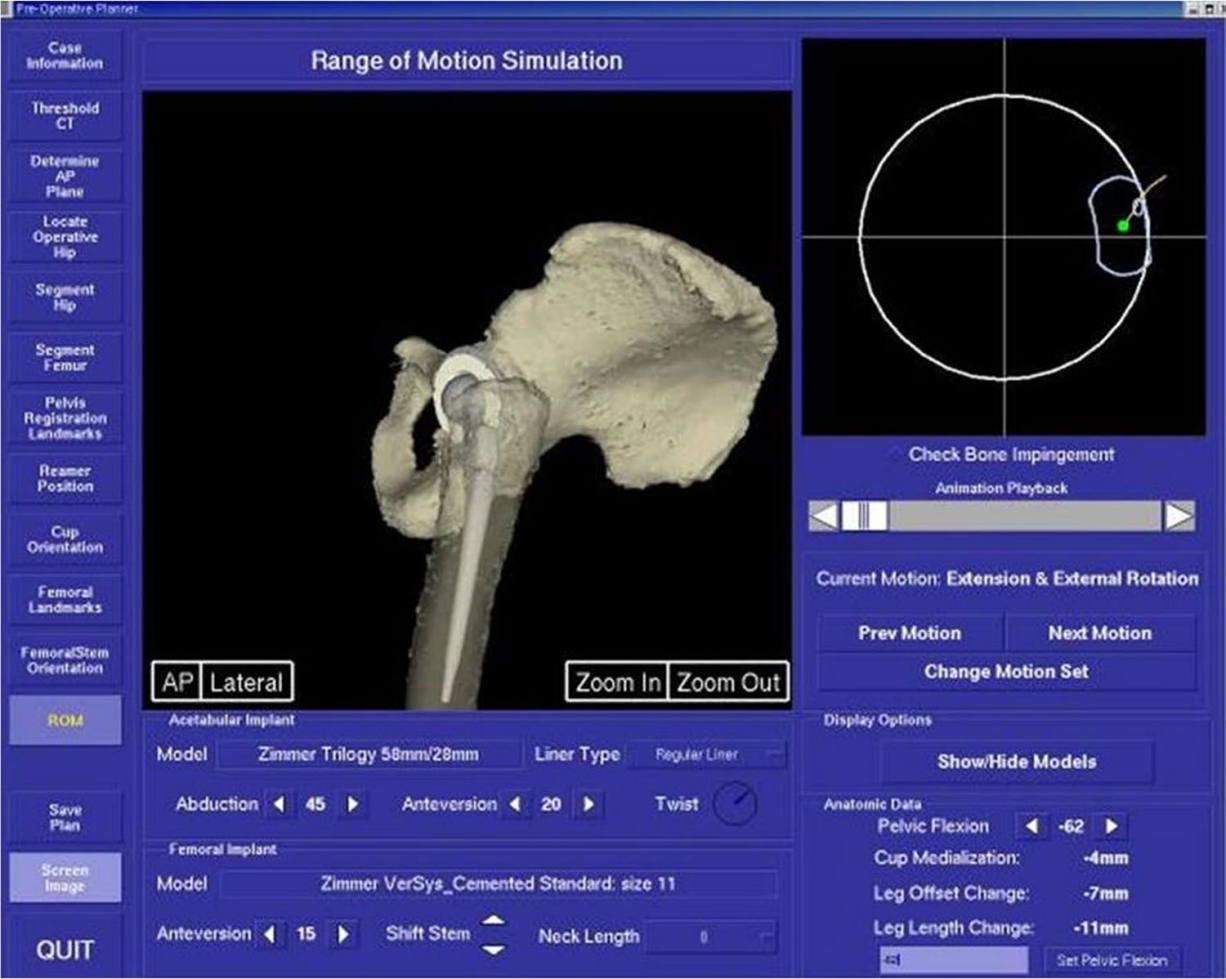
ROM simulator showing the flexion/extension of the pelvis and the corresponding ROM and impingement limits of THR

This study was conducted back in 2006. A range of motion (ROM) simulator was initially used to evaluate the clinical impact of the different patterns of PFE on ROM and impingement. The simulator was developed as part of an image-based navigation system to preoperatively plan the alignment of the femoral and acetabular components for individual patients. It displayed the ROM in real-time and calculated the positions of implant impingements for different leg positions (Fig. 1). For this study, the simulation maintained a constant implant alignment (relative to the APP), which has often been quoted as “optimal” (45° degrees abduction, 20° anteversion of the acetabulum, 15° anteversion for the femoral implant, 0 neck length, and 28 mm head). The implants used in the simulation were the Zimmer Versys femoral stem and Trilogy acetabular cup with a non-hooded liner. The only variable that was input to the simulator was the tilting of the pelvis to different degrees of flexion or extension to represent different patterns of pelvic orientations during standing and sitting.

The data obtained from our previous study were further analyzed to identify the pattern of PFE for every individual patient and group these patients accordingly into different categories [33]. In that study, prospectively collected lateral pelvic x-rays in the standing and sitting positions were obtained pre-operatively and three months postoperatively following THA of 84 patients. All patients underwent unilateral THA for osteoarthritis. To avoid uncontrolled variables (e.g., complications after bilateral THA) that may increase the risk of bias, patients with bilateral involvement were excluded.

The radiograph technique used was the one described by McCollum and Gray [11], where the patient is positioned with the affected hip next to the cassette in the standing and then in the sitting position in a straight-backed chair. One observer (MAH) measured the PFE from the lateral radiographs using the APP as a reference.

The term “pelvic orientation” is used to describe the alignment of the pelvis (e.g., flexion or extension), and the term “pelvic tilting” to describe the range of pelvic movement as the individual moves from standing to sitting. Pelvic flexion is the anterior alignment of the pelvis where the APP lies anterior to the vertical line, i.e., the APP angle here is positive. The pelvic extension is described when APP lies posterior to the vertical line, i.e., the APP angle is negative (Fig. 1).

To represent all possibilities of PFE, we moved the pelvis from the measured extreme flexion (+ 27°) to the measured extreme extension (− 64°) in 5° increments. When impingement occurred at certain degrees of pelvic flexion, the cup anteversion was adjusted to test the effects of increased and decreased anteversion on impingement.

The mean, standard deviation (SD), and range were calculated for all measurements. The statistical analysis was done using Microsoft Excel.

## Results

**Fig. 2:**
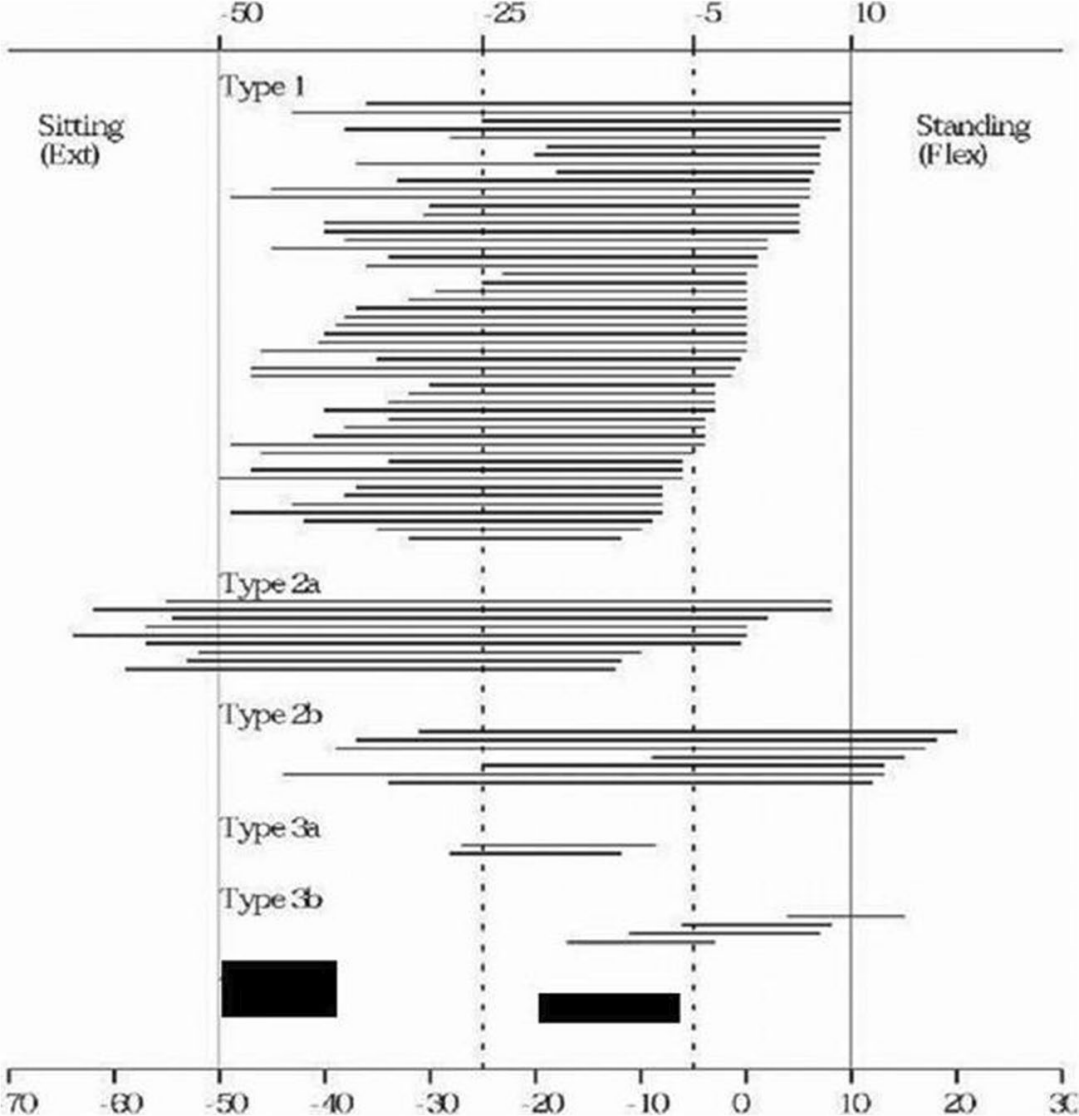
A chart shows different groups of patients according to the pattern of their pelvic orientation. Each transverse line represents an individual patient with the APP angle in standing (right) and in sitting (left). The length of the line is the range of pelvic tilting (ROM). The longitudinal lines are based on mean APP angles ± one standard deviation in standing and sitting. The outside solid lines are set as the hypermobility limits and the inner dotted lines are the stiffness limits

The mean age of patients was 62 years (range, 37 to 81), 40 females and 44 males. The affected hip was the right one in 55 patients and the left one in 29 patients. The mean body weight was 80 kg (SD, 19; range, 43 to 136). The mean height was 169 (SD, 10; range, 147 to 206). The analysis of the data of PFE and tilting for each patient is displayed in Fig. 2. Patients were classified into different groups, those in type I and II had a range of pelvic tilting (from standing to sitting) ≥ 20° and those in type III had a range of pelvic tilting < 20° (stiff pelvis). Patients in type I (normal pelvis) had their APP angles within the normal mobility limits in standing (+ 10°) and in sitting positions (− 50°). These mobility limits are defined by the mean plus one standard deviation of the APP angles in standing and sitting positions. Patients in type II (hypermobile pelvis) had their APP angles beyond the mobility limits either in standing (flexion type) or in sitting (extension type). Hence, a classification system was developed, based on the above findings, and supported by the ROM simulator testing (Fig. 2) (Table 1).

**Table 1:** Classification of PFE with suggestions for acetabular cup alignment

	Type I	Type II		Type III	
Name	Normal	Hypermobile pelvis (exaggerated response)		Stiff pelvis	
	Mobile	II-A extension type	II-b flexion type	III-a extension type	III-b flexion type
PFE in standing	Flexion	Flexion	Excessive flexion	Lack of flex	Flexion
PFE in sitting	Extension	Excessive extension	Extension	Extension	Lack of extension
Stability	Stable	Stable but sensitive to malalignment		Unstable even with optimal acetabular alignment	
		Weak anteriorly	Weak posteriorly	Anterior	Posterior

## Type I: normal mobile pelvis

Normally, the pelvis assumes an upright (APP angle is close to zero) position when patients are standing. This provides adequate anterior coverage of the acetabulum, increasing the stability of the hip when the leg is in extension. During sitting, the pelvis tilts backward to provide more posterior coverage. In this group, the cup could be positioned according to the quoted figures for abduction and anteversion. This type is relatively less sensitive to implant malposition errors.

## Type II: hypermobile pelvis

### Type II-a: hypermobile in extension

In these patients, the pelvis moves to extreme degrees of extension during sitting providing more posterior coverage but at the expense of anterior coverage. These patients are stable in positions of ADL but may be more prone to anterior dislocation in sitting if they externally rotate the operated leg. These patients may be more sensitive to mild degrees of increased cup anteversion. The acetabular cup could be aligned according to the recommended guidelines for THA. Excessive acetabular or femoral implant anteversion should be avoided.

### Type II-b: hypermobile in flexion

**Fig. 3:**
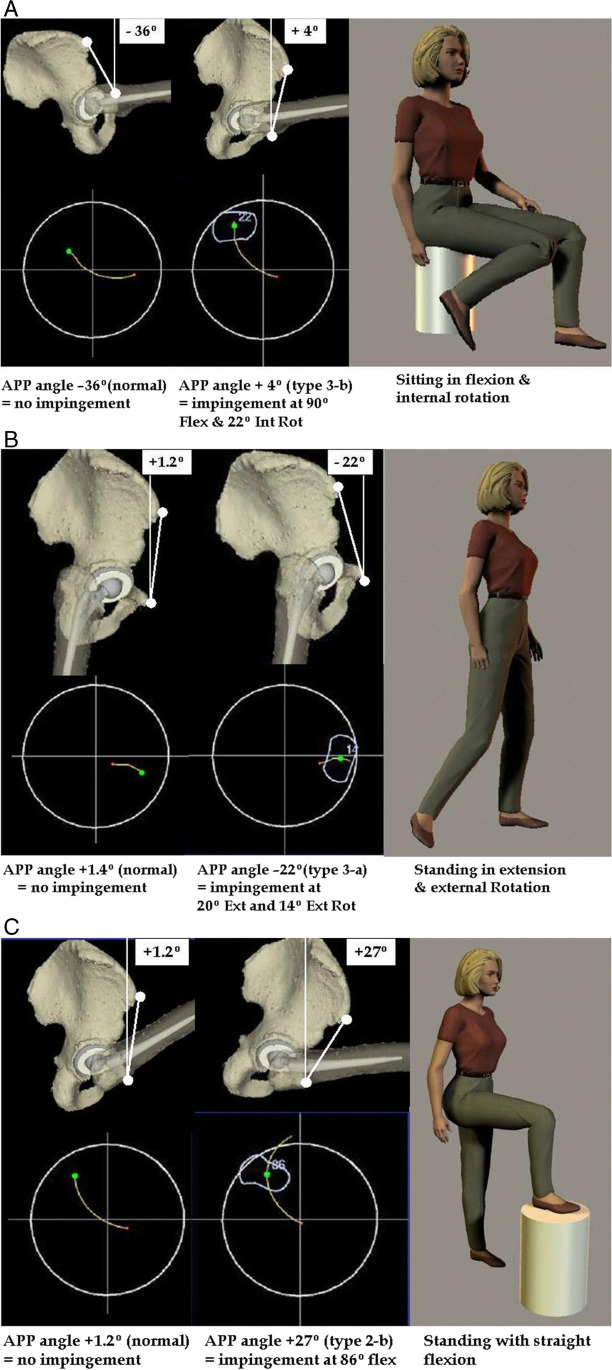
ROM simulator demonstrates the clinical impact of the different types of pelvises on ROM and impingement in THR. It compares patients with normal pelvis (type 1) and patients with abnormal pelvis who had impingements even in the presence of the quoted optimal implant alignment (abduction 45°, flexion 20°). **A** Type III-b patient (APP + 4°). **B** Type III-a patient (APP − 22°). **C** Type II-b patient (APP + 27°)

In these patients, the pelvis moves to extreme degrees of flexion during standing, providing more anterior coverage but at the expense of posterior coverage. These patients are stable in positions of ADL but prone to posterior dislocation in standing if they flex the operated leg combined with some internal rotation (Fig. 3B). These patients may be more sensitive to mild degrees of decreased cup anteversion. Acetabular and femoral implants could be aligned according to the recommended guidelines for THA. Excessive acetabular or femoral implant retroversion should be avoided.

## Type III: stiff pelvis

### Type III-a: stiff in extension

In a smaller group of patients, the pelvis is aligned extension, but it is stiff and does not significantly return to neutral upright alignment during standing, making a deficient anterior coverage. These patients are prone to anterior dislocation during standing particularly with extension and external rotation (Fig. 3B). Stability can be improved by decreasing the anteversion of the acetabular and/ or femoral implants.

### Type III-b: stiff in flexion

In this subgroup, the pelvis is stiff in flexion and does not significantly extend during sitting, resulting in less posterior coverage. These patients are prone to posterior dislocation during sitting activities particularly when the leg is in internal rotation (Fig. 3A). In these cases, the acetabular cup should be aligned in more anteversion.

## Further patient evaluation

Preoperative and three month postoperative clinical examination of the hip joints of a random sample of seven patients, representing different types of PFE showed no correlation between the hip ROM and patterns of pelvic orientation. No reports of dislocation or signs of instability were reported in this sample. The ROM simulator demonstrated the clinical impact of different patterns of PFE on ROM and impingement. The simulator performed the anterior and posterior stability test for implant impingement as follows: (A) straight flexion up to 130°, (B) flexion up to 90° followed by internal rotation up to 70°, and (C) extension of 30° followed by external rotation up to 70° (Fig. 3).

Figure 3A contrasts the differences in the sitting position between type I (normal mobile pelvis) and type III-b (stiff pelvis in flexion). The type I pelvis had an APP angle of − 36° (mean angle in sitting position). In this situation, with the hip flexed to 90 and internally rotated to 70°, no impingement occurred. In contrast, type III-b had an APP angle of + 4. This time, impingement occurred at 90° flexion and 27° internal rotation. Figure 3B contrasts the differences in the standing position between type I (normal mobile pelvis) and type III-a (stiff pelvis in extension). The type I pelvis had an APP angle of + 1.4° (mean angle in standing). In this situation, with the hip extended 20° and externally rotated 30°, no impingement occurred. In contrast, type III-a had an APP angle of − 22. This time, impingement occurred at 20° extension and 14° external rotation. Figure 3C contrasts the differences in the standing position between type I (normal mobile pelvis) and type II-b (hypermobile pelvis in flexion). The type I pelvis had an APP angle of + 1.4° (mean angle in standing). In this situation, with the hip flexed up to 110°, no impingement occurred. In contrast, type II-b had an APP angle of + 27°. This time, impingement occurred at 86°.

The results showed a trend for upright alignment of the pelvis in the standing position with a mean APP angle of + 1.2° (SD 7.9°, range –°22° to + 27°) and a tendency for posterior tilting in sitting positions with a mean APP angle of – 36.2° (SD 12.8°, range – 64° to + 4°). There was a very wide range of pelvic tilting for individual patients as they moved from the standing to sitting positions with tilting as low as 5° (stiff) for some patients, and as high as 70° (mobile) for others.

## Discussion

Dislocation is a common complication after THA and the rate of hip revision for instability is increasing [1–4]. Malalignment of the acetabular component has been identified as a major contributing factor to instability following THA [8–11, 34]. However, dislocation can still occur even with “optimal” implant alignment [12]. Recently, investigators studied the variation of PFE during daily activities and its effects on the acetabular cup version proposing the concept of “functional alignment” [14, 15]. Functional implant alignment is defined as the combination of the actual alignment of the implant in the bone and the flexion/extension of the pelvis in relation to the femur.

Nishihara et al. used image matching with CT scans to calculate pelvic flexion angle from anteroposterior radiographs in supine, sitting, and standing positions [30]. They measured the pelvic tilting from supine to sitting, then from supine to standing, and accordingly divided their patients into three groups. The combination of these two measurements resulted in a further classification of their patients into four groups. The classification was not clinically based, and no attempt has been made to quantify the effects of the variations of pelvic tilting on the stability of THA.

In our study, we showed that different patterns of PFE could influence the acetabular version and impingement following THA. The proposed classification system demonstrates the variations in pelvic orientation and its effect on functional acetabular alignment. This classification scheme is clinically based, and it is based on our data and the ROM simulator testing. In addition, this classification is supported by the clinical observations of other authors [17, 18]. For example, Tang et al. attributed the anterior instability and dislocation in patients with ankylosing spondylitis to the hyperextension position of the pelvis (type III-a in our series), which brought the acetabular cup to a more open position (anteversion). They postulated that if the cup is inserted according to the anatomy of the acetabulum, it becomes abnormal when the patient assumes an upright position with the pelvis rotated. They also questioned why these patients had anterior dislocation while the surgical approach was posterior [17]. Furthermore, Fijishiro et al. reported a case of recurrent anterior instability of THA due to the posterior inclination of the pelvis associated with lumbar kyphosis [35]. The mechanism of dislocation here could be explained by our proposed classification. The instability occurred because of the reduced anterior coverage caused by the hyper-extended pelvis even in the presence of apparently accurate anatomic alignment of the acetabular cup and regardless of the location of the surgical approach.

The proposed classification system is clinically useful and practical. It can help in understating the effects of PFE on the stability following THA. This approach can guide surgeons to preoperatively identify at-risk patients and plan a strategy to adjust implant alignment. Considering these factors, this system can be useful in planning for primary THA and in providing additional information for the management of unstable THA and the planning for revision surgery in those with recurrent dislocations. There may also be a prognostic value in predicting the stability after THA based on the type of pelvic tilting. The classification system can also guide the use of more stabilizing cups like the dual-mobility articulations, which have been proven to stabilize high dislocation risk hips of patients [36, 37].

Our study has a limitation; the ROM simulator used a generic pelvis to simulate the PFE and assessed implant but not bony impingement. The latter would require importing the CT scan of every patient to have a patient-specific pelvis and femur that would allow testing of bony impingement. This can be done in future studies. The differences in PFE angles have been correlated to the postoperative clinical conditions of individual patients but this would be considered in further investigations.

## Clinical relevance

The study showed that PFE was favorable in most THA patients (88% types I and II), and there was a smaller subset of patients with abnormal PFE who were susceptible to impingement and therefore possibly at higher risk of dislocation. Those patients may require adjustment of their acetabular and femoral implant alignment, which could be very different from the quoted “optimal” alignment. For example, patients who do not tilt their pelvis posteriorly during sitting or anteriorly in a standing or supine position may be at increased risk for instability. Also, patients who have extreme APP angles either in sitting or standing are more sensitive to malpositioning of the implant than patients with normal APP angles. The clinical importance of understanding the phenomenon of PFE should lead to the concept of “functional” acetabular alignment. These changes in PFE show that optimal acetabular alignment is a “moving target,” and surgeons may need to consider this in planning for implant alignment during THA. In addition, there may not be a single optimal implant alignment as is commonly quoted in the literature. The methods used in this study apply to any hospital facility or doctor’s office, as it only requires a preoperative lateral pelvic x-ray.

The concept of “functional” implant alignment may prove to be useful in the management of unstable THA as well, especially when there is no obvious implant malalignment. Morrey has emphasized the importance of defining the precise cause of the instability to plan the surgery that best addresses the particular problem [38]. Some authors classified the causes of instability into malrotation of components, soft tissue laxity, impingement, or multiple and unknown [39]. The findings of variable pelvic orientations and their effects on stability could explain these unknown causes of instability. Adjustment of cup position based on pelvic orientations in this group of patients may improve the treatment outcome of instability. Computer-assisted systems have the potential to plan, simulate, and implement the required alignment of the implants based on the individual pattern of pelvic orientation.

## Data Availability

All raw data used in the generation of this manuscript can be obtained by contacting the corresponding author (Mahmoud A Hafez).

## Abbreviations

ADL: Activities of daily life
APP: Anterior pelvic plane
ASIS: Anterior superior iliac spines
PFE: Pelvic flexion/extension
ROM: Range of motion
THA: Total hip arthroplasty
